# Immune-endothelial crosstalk at the nexus of neuroinflammation and vascular dysfunction in aging

**DOI:** 10.3389/fimmu.2026.1972284

**Published:** 2026-10-19

**Authors:** Zhitao Cui, Huaiyu Sun, Zhiqing Chen, Jiaai Li

**Affiliations:** 1 Department of Geriatrics, The First Hospital of Jilin University, Changchun, Jilin, China; 2 Department of Neurology, The First Hospital of Jilin University, Changchun, China; 3 Department of Neurology, Beijing Tiantan Hospital, Capital Medical University, Beijing, China; 4 Human Brain and Tissue Bank, China National Clinical Research Center for Neurological Diseases, Beijing Tiantan Hospital, Capital Medical University, Beijing, China

**Keywords:** blood–brain barrier, brain aging, endothelial dysfunction, immune–endothelial crosstalk, inflammaging, neuroinflammation, neurovascular unit

## Abstract

Aging is the major biological risk factor for cognitive decline and neurodegenerative disease. Increasing evidence indicates that neuroinflammation and cerebrovascular dysfunction are not independent consequences of aging but interact through the neurovascular interface. Cerebrovascular endothelial cells are central to this interaction because they form the blood–brain barrier (BBB) while actively regulating vascular tone, molecular transport, leukocyte trafficking, and inflammatory signaling. Here, we review how aging simultaneously primes immune and endothelial compartments through microglial sensitization, astrocytic remodeling, systemic inflammaging, endothelial senescence, altered transcytosis, oxidative stress, and senescence-associated inflammatory signaling. Immune-derived cytokines, reactive oxygen species, matrix metalloproteinases, complement signals, and inflammasome-related pathways can impair endothelial homeostasis, whereas aged endothelial cells relay systemic inflammatory signals through adhesion molecules, chemokines, complement receptors, and altered barrier transport. These reciprocal interactions can destabilize BBB function, cerebral blood flow regulation, and the broader neurovascular unit. Importantly, physiological aging should not be equated with neurodegenerative disease; rather, it may establish a compensated but vulnerable immune–endothelial state that is amplified by disease-specific modifiers. We discuss competing immune-first, vascular-first, and parallel-aging models and evaluate emerging therapeutic strategies aimed at restoring neurovascular homeostasis.

## 1 Introduction

Aging is the strongest biological risk factor for many chronic disorders, including Alzheimer’s disease (AD), Parkinson’s disease (PD), and vascular cognitive impairment and dementia (VCID) (1–3). Increasing evidence indicates that age-related cognitive decline cannot be explained solely by neuronal pathology. Instead, changes in cerebral vascular function and chronic neuroimmune activation converge within the neurovascular unit (NVU), where endothelial cells (ECs), pericytes, astrocytes, microglia, neurons, and extracellular-matrix components cooperate to maintain cerebral homeostasis (4–6).

Cerebrovascular ECs occupy a particularly important position because they form the principal cellular component of the blood–brain barrier (BBB). The BBB is not simply a static physical barrier: it regulates molecular transport, metabolic exchange, vascular signaling, and immune surveillance and therefore constitutes an active interface between the systemic circulation and the central nervous system (CNS) (7–9). Human dynamic contrast-enhanced magnetic resonance imaging has demonstrated age-associated increases in BBB permeability in the hippocampus, particularly in the CA1 and dentate gyrus, with greater disruption in individuals with mild cognitive impairment (10). Importantly, this finding suggests regional vascular vulnerability during aging rather than uniform failure of the entire BBB.

In parallel, aging remodels the immune compartment and cerebral vasculature. Microglia acquire altered response thresholds, astrocytic states change, and systemic low-grade inflammation develops, whereas cerebral ECs undergo oxidative stress, altered transport, impaired vasoregulatory signaling, and cellular senescence (11–25). These observations suggest that neuroinflammation and vascular dysfunction are not merely parallel consequences of aging. Rather, reciprocal immune–endothelial communication may convert initially compensated alterations into a self-amplifying neurovascular process. We therefore focus on how aging primes both sides of this interface, how bidirectional signaling destabilizes neurovascular homeostasis, and how this state may progress from physiological aging toward neurodegenerative disease. We therefore focus on how aging primes both sides of this interface, how bidirectional signaling destabilizes neurovascular homeostasis, and how this state may progress from physiological aging toward neurodegenerative disease (**Figure 1**).

**Figure 1:**
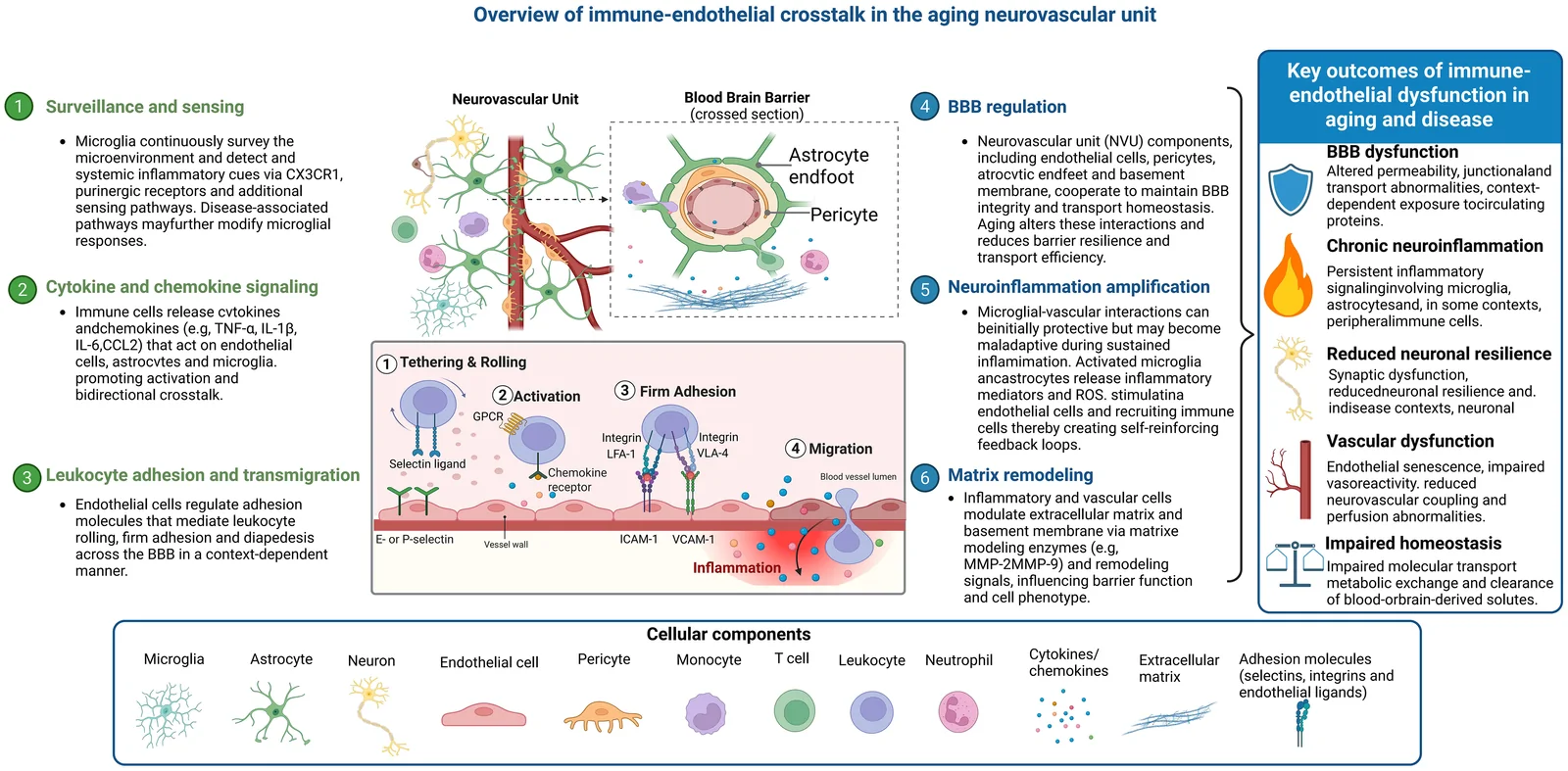
Overview of immune-endothelial crosstalk in the aging neurovascular unit. Aging remodels immune and cerebrovascular compartments, creating an altered immune-endothelial interface within the neurovascular unit (NVU). Microglial sensing, cytokine and chemokine signaling, leukocyte-endothelial interactions, and extracellular matrix remodeling collectively contribute to bidirectional communication between immune cells and cerebrovascular endothelial cells, influencing blood-brain barrier (BBB) integrity and neuroinflammatory responses. Dysregulated immune-endothelial interactions may contribute to age-associated BBB dysfunction, impaired neurovascular regulation, altered cerebral perfusion, and chronic neuroinflammation, potentially increasing vulnerability to neurodegenerative diseases. The schematic illustrates an integrated conceptual framework rather than a single linear causal pathway.

## 2 Aging primes the immune–endothelial interface

Aging produces substantial remodeling of innate and adaptive immunity. Chronic low-grade systemic inflammation, commonly termed inflammaging, arises from multiple interacting processes, including immunosenescence, metabolic dysfunction, cellular senescence, and persistent innate immune stimulation (11, 12). In the brain, microglia do not undergo uniform ‘activation.’ Rather, aged microglia acquire heterogeneous transcriptional and functional states and frequently become more responsive to subsequent inflammatory challenges, a phenomenon commonly described as microglial priming (13, 14). Astrocytes likewise display age- and context-dependent reactive states that can modify inflammatory signaling, metabolic support, and vascular homeostasis (15). Peripheral adaptive immunity also changes with age; for example, clonally expanded T cells have been identified within old neurogenic niches, illustrating that immune access to the aging CNS can be anatomically restricted but biologically meaningful (16, 17).

The vascular compartment undergoes complementary changes. Human and animal studies demonstrate age-associated alterations in BBB structure and molecular phenotype (18, 19). Single-cell RNA sequencing identified a population of senescence-associated cerebromicrovascular ECs in aged mouse brain (20), while brain ECs were shown to be highly responsive to age-related circulating factors, with capillary ECs exhibiting particularly prominent transcriptional changes (21). Vascular cell senescence can impair BBB integrity experimentally (22), and more recent work provides evidence that brain endothelial senescence contributes directly to age-related BBB dysfunction (23). Cellular senescence may further alter vascular–immune communication through the senescence-associated secretory phenotype (SASP), although SASP composition varies substantially according to cell type, stimulus, and tissue context (24, 25).

These findings support a model in which aging does not necessarily produce immediate neurovascular failure. Instead, it establishes a primed immune–endothelial interface characterized by reduced reserve and increased susceptibility to inflammatory, metabolic, vascular, and neurodegenerative stress (**Figure 2**).

**Figure 2:**
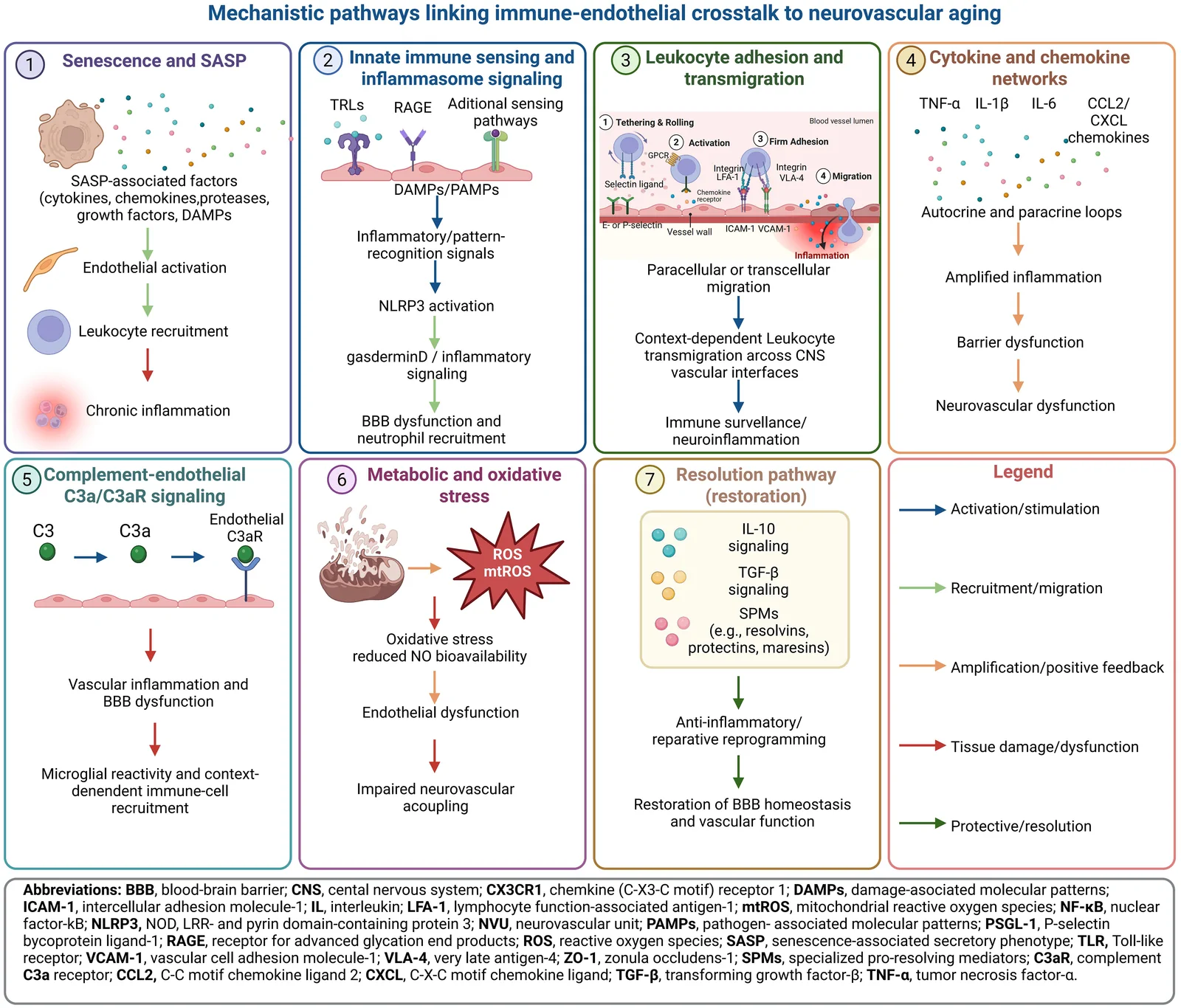
Mechanistic pathways underlying immune-endothelial crosstalk during neurovascular aging. Aging-associated immune-vascular interactions are mediated through multiple interconnected mechanisms. Cellular senescence and the senescence-associated secretory phenotype (SASP), innate immune sensing and inflammasome activation, leukocyte adhesion and transmigration, cytokine and chemokine signaling, endothelial complement C3a/C3aR signaling, oxidative stress, and impaired metabolic homeostasis collectively promote endothelial activation, BBB dysfunction, neurovascular impairment, and chronic neuroinflammation. In parallel, endogenous resolution pathways, including IL-10, TGF-β, and specialized pro-resolving mediators (SPMs), may counterbalance excessive inflammatory signaling and support restoration of BBB integrity and neurovascular homeostasis. The pathways shown are interconnected and context-dependent rather than strictly sequential.

## 3 Bidirectional immune–endothelial crosstalk

### 3.1 Immune-to-endothelial signaling

Immune activation can alter cerebrovascular EC phenotype through several overlapping mechanisms. Pro-inflammatory cytokines, including interleukin-1β and tumor necrosis factor-α, activate NF-κB and mitogen-activated protein kinase pathways and increase endothelial inflammatory gene expression and adhesion molecules (26–30). Oxidative stress provides a parallel route: reactive oxygen species generated by activated microglia, infiltrating leukocytes, NADPH oxidases, or dysfunctional mitochondria can alter endothelial nitric oxide bioavailability, cellular metabolism, and junctional organization (31, 35, 36). Matrix metalloproteinases (MMPs), particularly MMP-2 and MMP-9, can additionally remodel junction-associated proteins, basement membrane, and extracellular matrix, promoting barrier destabilization (28, 32–34).

Inflammasome signaling provides a useful example of context-dependent immune-to-vascular amplification. NLRP3 activation contributes to amyloid- and tau-associated pathology in experimental AD models (37–39), whereas gasdermin D mediates pore formation downstream of inflammasome activation (40). More recently, repeated peripheral inflammatory challenge in mice was shown to activate microglial NLRP3–gasdermin D signaling and promote BBB dysfunction and neutrophil recruitment (41). This finding should not, however, be interpreted as evidence that NLRP3 universally initiates BBB aging; the experimental context involved peripheral inflammation rather than uncomplicated physiological aging.

Microglia themselves can also exert temporally opposing vascular effects. During systemic inflammation, vessel-associated microglia initially contributed to barrier protection but became detrimental during sustained inflammatory exposure, in part through interactions with astrocytic endfeet (42). Pattern-recognition pathways provide further routes through which danger-associated signals can amplify such responses (43). Thus, immune activation should not be regarded as uniformly injurious. Its vascular consequences depend on intensity, duration, anatomical context, and disease stage.

### 3.2 Endothelial-to-immune signaling

The reverse direction is equally important and provides one of the strongest arguments for considering the endothelium an active immunological interface. Aged brain ECs can reshape immune activity through adhesion molecules, complement receptors, chemokines, SASP-related factors, and altered transport.

A particularly compelling example is vascular cell adhesion molecule 1 (VCAM-1). Aged circulation induces an inflammatory phenotype in brain ECs, including focal endothelial VCAM-1 upregulation. In mice, antibody blockade or endothelial deletion of Vcam1 attenuated microglial reactivity and several detrimental effects associated with aged circulation (44). This provides direct evidence that the endothelium can translate systemic aging signals into CNS immune responses.

Complement signaling represents another mechanistic bridge. Endothelial C3a receptor signaling increases during aging and is associated with vascular inflammation, BBB permeability, peripheral lymphocyte infiltration, and microglial reactivity. Endothelial-specific C3ar1 deletion attenuated several of these phenotypes, supporting direct vascular–immune crosstalk (45).

Aging also modifies endothelial transport rather than merely producing nonspecific barrier ‘leakiness.’ Physiological plasma-protein transport shifts from ligand-specific receptor-mediated transport toward less selective caveolar transcytosis in aged mice, accompanied by altered pericyte coverage (46). Fractalkine/CX3CR1 signaling, broader chemokine networks, sphingosine-1-phosphate signaling, and endothelial heterogeneity provide additional context-dependent routes for communication among vascular, resident immune, and circulating immune compartments (47–53).

The aging endothelium can therefore be viewed as a translator of systemic inflammatory information: it detects circulating changes and converts them into altered adhesion, transport, inflammatory signaling, and immune-cell behavior.

### 3.3 BBB dysfunction, immune trafficking, and neurovascular amplification

Peripheral immune surveillance of the CNS involves anatomically distinct interfaces and highly regulated leukocyte–endothelial interactions (54). BBB disruption can additionally expose brain tissue to blood-derived proteins such as fibrinogen, which themselves have pro-inflammatory effects (55, 56). Nevertheless, physiological aging should not be equated with massive generalized leukocyte invasion. Available evidence instead supports region-, niche-, and context-dependent changes in immune trafficking.

Immune–endothelial dysfunction also propagates through the broader NVU. Pericyte loss can impair both BBB integrity and cerebral perfusion and may precede secondary neuronal and inflammatory abnormalities in experimental models (57–59). Aging and AD are associated with impaired neurovascular coupling, involving endothelial dysfunction, oxidative stress, and altered nitric oxide signaling (60–63). Hypoxia-responsive pathways, including HIF signaling, may provide an additional context-dependent modifier of cerebrovascular adaptation (64). Astrocytes can further influence BBB integrity and cerebral blood flow through endfoot signaling, vascular growth factors, and vasoactive pathways (65–67). These cell–cell relationships occur within a highly specialized BBB architecture governed by coordinated endothelial, pericytic, glial, and extracellular-matrix signaling (68, 69).

The resulting process is therefore not simply ‘inflammation causing BBB leakage.’ A more complete sequence is immune/endothelial priming → reciprocal inflammatory signaling → altered barrier transport and adhesion → impaired BBB and vascular regulation → glial and peripheral immune amplification → further neurovascular dysfunction.

## 4 From physiological aging to neurodegenerative disease

A critical distinction is that age-related immune–endothelial remodeling is not biologically equivalent to neurodegenerative disease. Physiological aging can involve microglial priming, endothelial senescence, altered transport, and reduced neurovascular reserve while remaining at least partly compensated. Disease-specific factors may subsequently amplify this vulnerable state.

In AD, APOE4 provides a clear example. Human studies demonstrate hippocampal and medial temporal BBB dysfunction in APOE4 carriers, including cognitively unimpaired individuals, and this vascular phenotype can occur independently of conventional amyloid-β and tau measures (70). Amyloid-β itself can influence vascular function through pericytes and capillary constriction (71), while human neuropathological studies demonstrate complex regional relationships among amyloid accumulation, hypoperfusion, BBB dysfunction, and pericyte markers (72). Neuroinflammation, adaptive immune responses, and cellular senescence further interact with these vascular mechanisms in AD (73–77).

VCID may represent a context in which a vascular-first sequence is particularly plausible. Human imaging demonstrates increased BBB permeability in vascular cognitive impairment, and MMP activity has been associated with barrier opening in this population (78, 79). Chronic cerebrovascular disease, small-vessel pathology, and hypoperfusion can therefore initiate or amplify inflammatory responses, although mixed vascular and AD pathology is common in older adults (80).

Evidence for BBB dysfunction also exists in PD. Postmortem studies demonstrate altered striatal BBB permeability (81), while dynamic contrast-enhanced MRI has identified increased BBB leakage in patients with PD, particularly in posterior white matter and several disease-relevant regions (82). However, compared with AD and VCID, the temporal and causal contribution of aging-specific immune–endothelial signaling to PD remains less well established.

Regional and cellular heterogeneity further complicates these comparisons. Single-nucleus analysis of human brain ECs has revealed pronounced region-specific transcriptional heterogeneity and distinct AD-associated endothelial responses (83), consistent with earlier demonstrations of vascular zonation (84) and regional susceptibility of hippocampal and white-matter systems during aging (85, 86). Recent reviews likewise emphasize that BBB aging encompasses multiple cellular components and processes rather than a single permeability phenotype (87).

Importantly, not all studies identify increased paracellular BBB leakage during normal aging. In aged mice, extensive transcriptional changes in barrier-related pathways were observed without measurable increases in paracellular permeability using several assays (88). This apparent discrepancy with human imaging studies is informative rather than contradictory: species, brain region, tracer, transport route, and measurement method all influence what is operationally defined as ‘BBB dysfunction.’ Endothelial senescence may similarly affect barrier properties, vascular remodeling, and inflammatory interactions in ways that differ across physiological aging and overt cerebrovascular disease (89).

Thus, aging may be best viewed as establishing a compensated but vulnerable neurovascular state, whereas AD, VCID, and PD add disease-specific modifiers that differ in magnitude, timing, and dominant initiating mechanisms. Key similarities and distinctions in immune–endothelial alterations across physiological aging, AD, VCID, and PD are summarized in **Table 1**.

**Table 1:** Immune–endothelial alterations across physiological aging and major neurodegenerative/vascular cognitive disorders.

Axis/process	Physiological aging	AD	VCID	PD	Therapeutic status
Microglial inflammatory responsiveness	Primed and heterogeneous states (13, 14)	Amplified and disease-associated states; causality is stage-dependent (37–39, 73–76)	Often secondary to vascular injury or hypoperfusion; mixed mechanisms (80)	Neuroinflammatory activation is reported, but immune–vascular causal ordering remains unclear	Predominantly preclinical; broad anti-inflammatory suppression may impair physiological immune functions
Endothelial senescence / SASP	Demonstrated in experimental models, with brain endothelial senescence linked to BBB dysfunction (20, 22–24)	Plausible contributor, but direct endothelial-specific evidence is less established than in physiological aging	Biologically plausible in vascular injury/cognitive impairment; direct causal evidence remains limited (89)	Limited direct evidence	Senolytic/senomorphic strategies are emerging; efficacy for brain endothelial dysfunction is unproven (96, 97)
VCAM-1-mediated signaling	Upregulated by aged circulation; endothelial VCAM-1 can relay systemic aging signals to microglia (44)	Potentially relevant, but disease-specific endothelial evidence is limited	Plausible in vascular inflammation; direct aging-linked evidence is limited	Limited evidence	Strong mechanistic mouse evidence in the aged-circulation paradigm (44)
Complement–endothelial signaling	Endothelial C3aR drives vascular inflammation and BBB permeability in aging mice (45)	Potential contributor; the endothelial-specific role is not yet established	Understudied	Understudied	Preclinical; endothelial C3aR has direct mechanistic support in aging mice (45)
BBB permeability / barrier function	Regional and assay-dependent; not uniformly increased with normal aging (10, 88)	Frequently abnormal, including early BBB dysfunction in susceptible regions and APOE4 carriers (70, 72, 102)	Prominent in clinically defined subsets; MMP-associated barrier opening has been reported (78, 79)	Reported in postmortem and DCE-MRI studies (81, 82)	Biomarker and therapeutic target under study; no established BBB-restoring therapy
BBB transcytosis	Age-related shift from ligand-specific receptor-mediated transport toward less selective caveolar transcytosis in mice (46)	Altered transport is likely, but disease-specific transcytosis mechanisms remain incompletely defined (102)	Poorly characterized	Limited evidence	Experimental
Peripheral immune interaction	Altered access and immune niches; not equivalent to generalized leukocyte invasion (16, 17, 54)	Increased immune surveillance/trafficking in selected CNS compartments (75)	Context-dependent recruitment may accompany vascular injury (78–80)	Emerging and heterogeneous evidence; extent and causality remain unclear	Context-dependent; indiscriminate blockade could impair immune surveillance
Pericyte / CBF dysfunction	Reduced neurovascular reserve, with age-related pericyte and neurovascular-coupling abnormalities (57–61)	Prominent; pericyte injury, capillary constriction and neurovascular dysfunction are well documented (62, 70–72)	Prominent vascular component of VCID pathophysiology (78–80)	Less well defined	Mostly preclinical
Dominant initiating model	Parallel aging with partial compensation	Mixed/bidirectional; vascular and immune pathways interact	Relatively vascular-biased	Uncertain	Mechanism- and stage-based stratification is likely required

The “dominant initiating model” row represents a conceptual synthesis of the available evidence rather than an established causal hierarchy.

AD, Alzheimer’s disease; BBB, blood–brain barrier; CBF, cerebral blood flow; C3aR, complement C3a receptor; DCE-MRI, dynamic contrast-enhanced magnetic resonance imaging; MMP, matrix metalloproteinase; PD, Parkinson’s disease; SASP, senescence-associated secretory phenotype; VCAM-1, vascular cell adhesion molecule 1; VCID, vascular cognitive impairment and dementia.

## 5 Therapeutic targeting of immune–endothelial crosstalk

Therapeutic manipulation of this axis is attractive because several components appear experimentally reversible. Resolution pathways, including specialized pro-resolving mediators, interleukin-10, and transforming growth factor-β signaling, may counterbalance chronic inflammatory signaling (90–93). Endothelial pathways such as angiopoietin–Tie and sphingosine-1-phosphate signaling provide additional potential approaches to barrier stabilization (94).

Preclinical experiments provide the strongest evidence to date. Endothelial VCAM-1 blockade can reduce microglial reactivity and improve age-related brain phenotypes in mice (44), endothelial C3aR deletion attenuates vascular inflammation and immune activation (45), and mitochondrial-targeted antioxidant treatment can restore endothelial-dependent neurovascular coupling in aged mice (61). Senescence-directed interventions are similarly attractive because senescent vascular and glial cells can contribute to age-associated pathology (23, 25). However, CNS delivery remains a substantial obstacle, motivating continued development of barrier-targeted delivery technologies (95).

Clinical translation remains much less mature. Senolytics have progressed from mechanistic geroscience concepts toward clinical investigation in AD and aging (96). A small 2025 pilot study of intermittent dasatinib plus quercetin in older adults at risk for AD provides early feasibility information but does not establish efficacy for cognitive decline or cerebrovascular endothelial dysfunction (97). More broadly, the history of AD drug development emphasizes the difficulty of translating biologically plausible pathways into effective therapies (98), while biomarker development will be essential for identifying appropriate intervention windows and patient subgroups (99).

Therapeutic suppression of immune–endothelial signaling also carries risks. Inflammation, endothelial activation, immune trafficking, and cellular senescence have physiological roles in host defense, tissue repair, and stress adaptation. The therapeutic goal should therefore be restoration of homeostatic immune–endothelial communication rather than indiscriminate suppression of inflammation.

## 6 Discussion and future perspectives

The major unresolved question is whether immune activation or vascular dysfunction initiates the age-associated cycle. Available evidence does not support a universal hierarchy. Systemic inflammatory challenges can produce an immune-first sequence, whereas pericyte loss, vascular risk, endothelial senescence, or hypoperfusion can generate a vascular-first pathway. A third possibility is increasingly compelling: common features of biological aging—including mitochondrial dysfunction, cellular senescence, altered circulating factors, metabolic dysregulation, and systemic inflammaging—may independently prime both compartments before reciprocal signaling begins.

Accordingly, initiation, amplification, and progression should be distinguished mechanistically. A pathway may not initiate aging pathology yet still be essential for converting compensated dysfunction into a self-sustaining state. Systemic factors, including age-related circulating proteins and gut-derived signals, may modify this threshold (100, 101), while BBB dysfunction and AD pathology may subsequently become mutually reinforcing (102–104).

Heterogeneity represents a second major challenge. Endothelial and microglial states vary across vascular segments, anatomical regions, age, sex, genetic background, and disease stage. Single-cell sequencing has become a useful framework for resolving disease-associated cellular and molecular heterogeneity in AD and PD (105). Transcriptomic integration and deconvolution analyses further show that microglial responses to ischemic–hypoxic injury differ across neonatal and adult contexts and evolve dynamically over time (106). These injury studies should not be equated with physiological aging, but they reinforce the broader point that microglial responses are context- and stage-dependent. Single-cell and spatial approaches should therefore move beyond the question of whether ‘microglia’ or ‘endothelial cells’ are activated and instead identify which cellular states interact, where, and at what stage. Longitudinal imaging combined with molecular biomarkers and cell-specific perturbation will be required to establish causal ordering in humans.

The emerging picture is consequently more nuanced than a simple vicious cycle. Aging appears to reduce neurovascular reserve while simultaneously increasing immune and endothelial responsiveness to stress. Reciprocal signaling can then amplify dysfunction, but the initiating event and dominant pathway vary with biological context. Contemporary models of neurovascular aging increasingly support this integrated framework (107).

In this view, immune–endothelial crosstalk is not simply another downstream consequence of aging. It is a dynamic interface capable of determining whether an aging neurovascular system remains compensated or progresses toward persistent inflammation, vascular failure, and neurodegeneration.
